# S100A4 contributes to colitis development by increasing the adherence of *Citrobacter rodentium* in intestinal epithelial cells

**DOI:** 10.1038/s41598-017-12256-z

**Published:** 2017-09-21

**Authors:** Jinhua Zhang, Ying Jiao, Shasha Hou, Tian Tian, Qi Yuan, Huaijie Hao, Zhenlong Wu, Xuexiang Bao

**Affiliations:** 1 College of Life Science and Bioengineering, Beijing Jiaotong University, Beijing, 100044 P.R. China; 2 School of Life Sciences, Northeast Normal University, Changchun, 130024 P.R. China; 3 CAS Key Laboratory of Pathogenic Microbiology and Immunology, Institute of Microbiology, Chinese Academy of Sciences, Beijing, 100101 P.R. China; 4 State Key Laboratory of Animal Nutrition, College of Animal Science and Technology, China Agricultural University, Beijing, 100193 P. R. China

## Abstract

S100A4 has been implicated in cancer and several inflammatory diseases, but its role in inflammatory bowel disease has not been well investigated. Here, upon infection with *Citrobacter rodentium*, a model for enteropathogenic *Escherichia coli* infection in humans, induced the infiltration of a large number of S100A4^+^ cells into the colon in wild type (WT) mice. Deficiency of S100A4 reduced weight loss, bacterial colonization and colonic pathology. Furthermore, the expression of inflammatory cytokines and the recruitment of macrophages and neutrophils also decreased significantly in S100A4 knock out (*S100A4* ^−/−^) mice. *In vitro*, soluble S100A4 directly up-regulated expression of integrin β−1 in intestinal epithelial cells and significantly increased the adherence of *C*. *rodentium* to intestinal epithelial cells. Additionally, the effects of S100A4 on the adherence of *C*. *rodentium* to epithelial cells could be abolished by a receptor for advanced glycation end products (RAGE)-specific inhibitor (FPS-ZM1). Therefore, these data indicate a novel mechanism for S100A4 that promotes colitis development by enhancing host adhesion and colonization of *Citrobacter rodentium* through the S100A4-mediated host inflammatory responses.

## Introduction

Enteropathogenic *Escherichia coli* (EPEC) and Enterohemorrhagic *Escherichia coli* (EHEC) are major causes of severe diarrhea and death worldwide^1^. They pose a significant public health risk, especially in developing countries where they contaminate food and water supplies^2^. EPEC causes infantile diarrhea, and the infection leads to dehydration and death^3^. EHEC is also a severe health threat, causing hemorrhagic colitis and hemolytic-uremic syndrome, a potentially fatal disease, and the infections happen particularly in young children, elderly people and immunocompromised individuals^4–6^. EPEC and EHEC, called attaching-and-effacing (A/E) pathogens, induce characteristic A/E lesions in the intestinal epithelium, which are important for establishing an infection in the host. They infect their hosts by intimately attaching to the surface of the intestinal epithelium and effacing the brush border microvilli^7^. Upon infection, A/E pathogens displace the commensal flora and cause intestinal inflammation characterized by crypt hyperplasia, goblet cell depletion, and damage to the epithelium. Additionally, infection with these pathogens induces infiltration of immune cells and edema within the lamina propria^8,9^. Both EPEC and EHEC are poorly pathogenic in mice, but *C*. *rodentium* is a Gram-negative A/E bacterium that specifically infects the mouse colon epithelial cells and causes damage to the epithelial layer^10,11^. Therefore, infection of mice with *C*. *rodentium* is an excellent *in vivo* model of colitis.

S100 proteins belong to a family of low molecular weight, EF-hand (E- helices and F-helices) calcium-binding proteins that regulate calcium-dependent and calcium-independent processes^11^. S100A4, also called fibroblast-specific protein 1, is a member of the S100 protein family. S100A4 was initially cloned in metastatic cells and fibroblasts, was identified as a metastasis promoter and has mainly been studied in relation to cancer^12^. It promotes motility and invasion of existing tumor cells, resulting in aggressive metastasis, and is expressed in various cell types, including fibroblasts, macrophages, and malignant cells^13–16^. Intracellularly, S100A4 binds to several targets regulating cytoskeletal dynamics and cell motility and proliferation^17^. Moreover, S100A4 is secreted from both tumor and non-malignant cells and exerts extracellular effects regulating cell mobility, invasion, and angiogenesis by interacting with annexin II, RAGE, and heparan sulfate proteoglycans^18–20^. In our previous study, we found an additional mechanism of action where S100A4^+^ cells promote 7, 12-dimethylbenz-(a)anthracene/ 12-O-tetradecanoylphorbol-13-acetate (DMBA/TPA) induced skin tumor development by promoting chronic inflammation^21^.

In fact, S100 proteins are associated with inflammatory responses. Expression of S100 proteins has been shown in arthritis and ulcerative colitis^22^. In addition, certain members, such as S100A7, S100A8 and S100A9, have the power to kill bacteria through modulating pH or inhibition of microbial growth^23,24^. It remains unclear whether S100A4 is involved in intestinal inflammation.

For this purpose, we used S100A4 knock out (*S100A4* ^−/−^) and wild-type (WT) mice orally challenged with *C*. *rodentium* to establish a model for studying the role of S100A4 and its related molecular mechanism in colitis. In the present study, we found that S100A4 contributes to bacterial colonization at sites of infection. The expression of S100A4 is up-regulated in *C*. *rodentium*-infected mouse colons. Infection-induced inflammation and colonic pathology are attenuated in *S100A4* ^−/−^ mice. Further mechanistic studies found that S100A4 increased the bacteria adherence to intestinal epithelial cells by up-regulating adherence molecular-integrin β-1 and directly promoted colonization.

## Results

### The expression of S100A4 is up-regulated in *C*. *rodentium*-infected mouse colons

**Figure 1:**
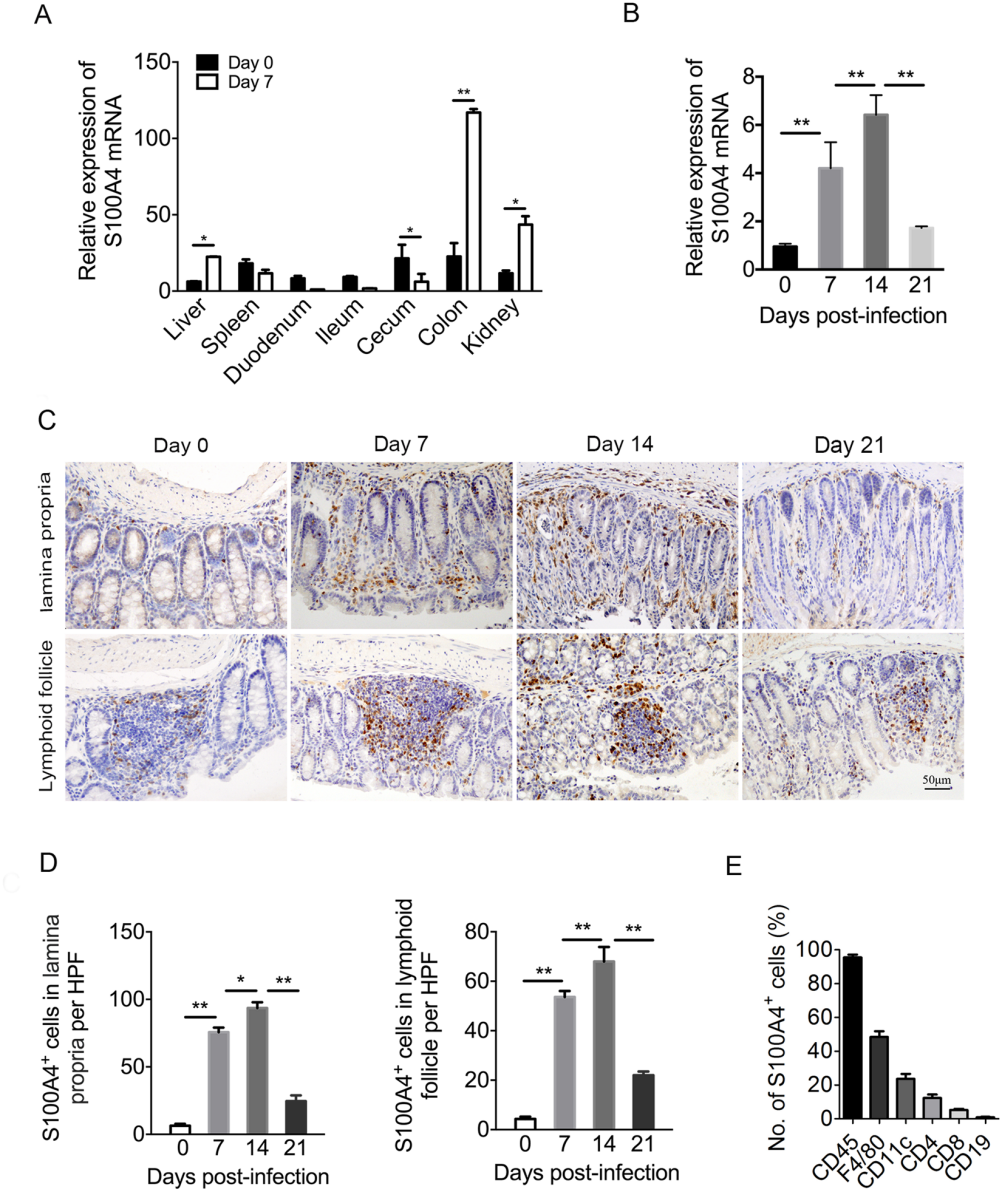
The expression of S100A4 is up-regulated in *C. rodentium*-infected mouse colons. C57BL/6 mice were infected orally with *C. rodentium* as described in the Materials and Methods. Colons and other tissues were collected at different time points. (**A**) Real-time quantitative PCR analysis of S100A4 mRNA in various tissues from infected C57BL/6 mice on day 0 and day 7 after infection. GAPDH was used as the reference control, (*n* = 4); *P < 0.05, **P < 0.01. The mRNA level of liver of non-infected mice is set as 1.00 to calibrate the relative levels in other tissues. (**B**) Real-time quantitative PCR analysis of S100A4 mRNA in colons from infected WT mice on day 0, 7, 14, 21 after infection. GAPDH was used as the reference control, (n = 4); **P < 0.01. The mRNA level of non-infected mice (day 0) is set as 1.00 to calibrate the relative levels in other days. (**C**) Immunohistochemical staining for S100A4 (brown) in colon sections from C57BL/6 mice on day 0 (uninfected) and day 7, 14, and 21 after infection (*n* = 4). (**D**) The numbers of S100A4^+^ cells per HPF ( × 200), (*n* = 4); *P < 0.05, **P < 0.01. The experiment was performed by an observer blinded to the experimental condition. (**E**) Flow cytometry analysis of the phenotypes of S100A4^+^ cells in the colons of S100A4^+/+^.^GFP^ mice after *C. rodentium* infection 7 days by staining GFP^+^ cells with CD45, F4/80, CD11c, CD4, CD8 and CD19 antibodies.

It is still not clear whether S100A4 is expressed in the mouse colon during *C*. *rodentium* infection. To investigate the kinetics of S100A4^+^ cells during this process, C57BL/6 mice were orally inoculated with 2 × 10^9^ CFU *C*. *rodentium* in 200 μl PBS. Various tissues were collected both prior to and during *C*. *rodentium* infection. The mRNA expression of S100A4 was examined by real time quantitative PCR. As shown in Fig. 1A, similar low levels of S100A4 mRNA in different tissues of uninfected WT mice were observed. However, high S100A4 mRNA levels were detected in colons after *C*. *rodentium* infection on day 7 (*P* < 0.01). The expression of S100A4 mRNA of mice colons on day 0, day7, day 14 and day 21 p.i. were further detected (Fig. 1B). The expression of S100A4 mRNA in mice colons was up-regulated on day 7 and day 14 p.i. (*P* < 0.01) and down-regulated on day 21 p.i. S100A4^+^ cells in colons at different time points were then stained by immunohistochemistry (IHC) (Fig. 1C). There were very few S100A4^+^ cells in the untreated colon. However, *C*. *rodentium* infection led to a rapid increase of these cells. There were more S100A4^+^ cells in the *C*. *rodentium* affected colon on day 7 than in untreated colon, which increased more on day 14. The number of S100A4^+^ cells decreased on day 21. Most S100A4^+^ cells accumulate in the lamina propria and lymphoid follicle in the colons (Figs. 1C,D) (P < 0.05, *P* < 0.01). Those consistent results clearly demonstrate that the S100A4 expression in the colons of WT mice was up-regulated during *C*. *rodentium* infection.

To identify the cellular source of S100A4 in the colon, S100A4^+/+^.GFP transgenic mice expressing green fluorescent protein (GFP) under the control of the S100A4 promoter were treated with DSS, and then cells were isolated from colon tissues, were co-stained with cellular marker antibodies for various cell types and were analyzed by flow cytometry. As shown in Fig. 1E, among the S100A4-GFP^+^ cells, approximately 96.5% were CD45^+^, mainly S100A4-GFP^+^ cells expressing myeloid cell markers, 48.4% were F4/80^+^ and 23.6% were CD11c^+^. In addition, a small number of the S100A4-GFP^+^ cells expressed markers of T cells, B cells and granulocytes (Fig. 1E). Double immunofluorescence staining of S100A4 and different cellular markers in the colon tissues showed similar results (Supplementary Fig. 1). In addition, S100A4^+^ cells seldom expressed α-SMA detected by double staining, showing that they were not fibroblasts (Supplementary Fig. 1).

### Infection-induced weight loss and *C*. *rodentium* colonization are attenuated in S100A4-deficient mice

**Figure 2:**
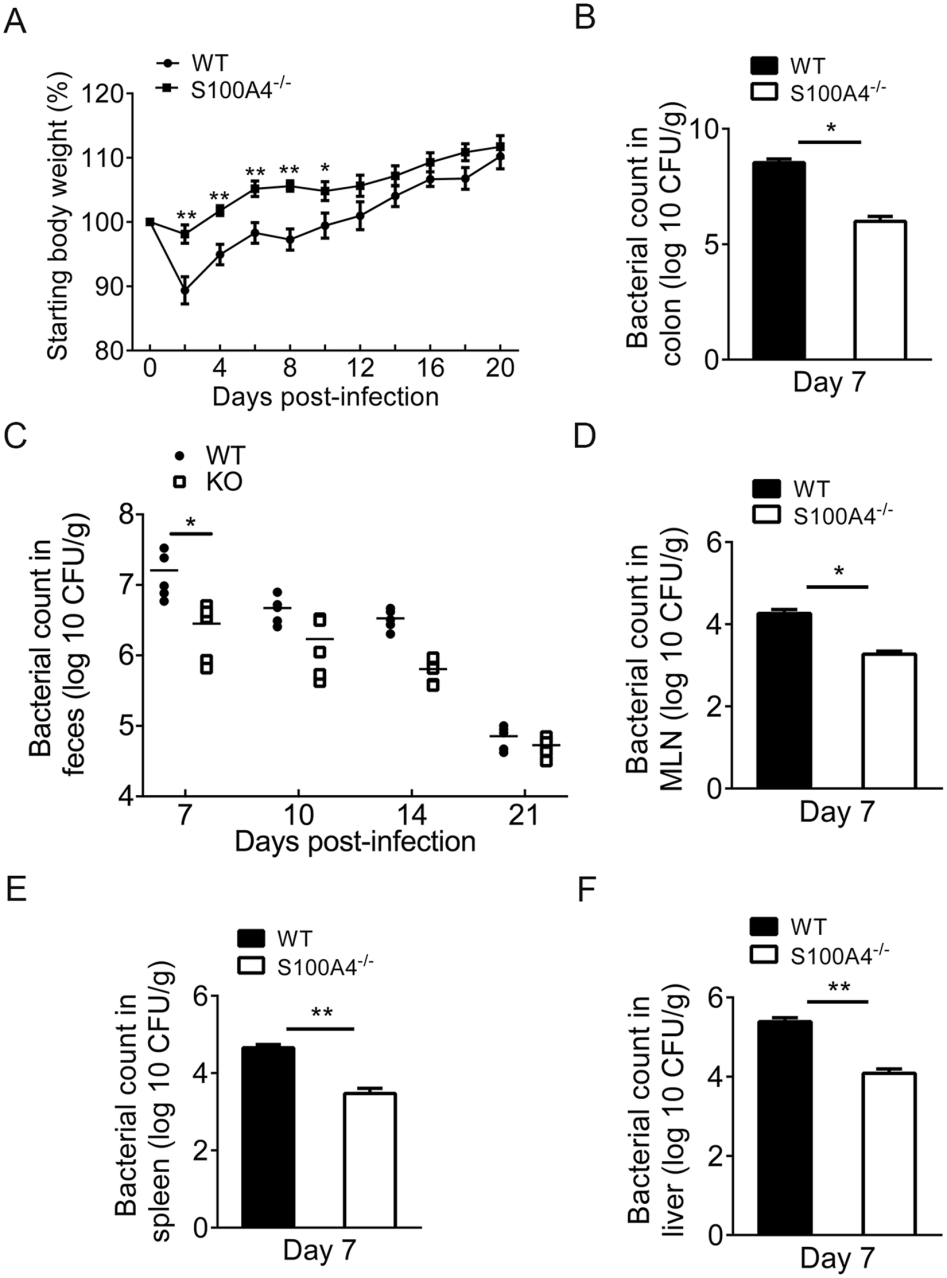
Infection-induced weight loss and *C. rodentium* colonization are attenuated in S100A4-deficient mice. Groups of WT and *S100A4* ^−/−^ mice were treated with *C. rodentium* for 3 weeks. (**A**) Body weight changes of infected WT and *S100A4* ^−/−^ mice are shown, (*n* = 5); *P < 0.05, **P < 0.01. (**B**, **D**–**F**) Bacterial titers in homogenates of colon, MLN, spleen and liver from WT and *S100A4* ^−/−^ mice on day 7 after infection (*n* = 5); *P < 0.05 and **P < 0.01. (**C**) Bacterial titers in fecal homogenates from WT and *S100A4* ^−/−^ mice at different time points after *C. rodentium* infection (*n* = 5); *P < 0.05.

To determine the role of S100A4 in the host response to an A/E pathogen *in vivo*, both *S100A4* ^−/−^ mice and WT mice were orally infected with 2 × 10^9^ CFU *C*. *rodentium* in 200 μl PBS. Infection-induced weight loss in mice was monitored for 20 days. As shown in Fig. 2A, weight loss in *S100A4* ^−/−^ mice was minimal and significantly less than that seen in WT mice on days 2, 4, 6, 8 (*P* < 0.01) and 10 (*P* < 0.05). Furthermore, *C*. *rodentium* colonization of colons in *S100A4* ^−/−^ mice was significantly less than those in WT mice after infection on day 7 (*P* < 0.05) (Fig. 2B). In addition, *S100A4* ^−/−^ mice had significantly lower fecal counts of *C*. *rodentium* on day 7 p.i. (*P* < 0.05) (Fig. 2C) and lower systemic infection in the mesenteric lymph nodes (MLN) (*P* < 0.05), spleen (*P* < 0.01) and liver (*P* < 0.01) on day 7 p.i. (Fig. 2D–F). Collectively, our data suggested that the counts of *C*. *rodentium* were reduced in *S100A4* ^−/−^ mice on day 7 p.i. compared with WT mice. However, there was a higher bacterial count in WT mice, suggesting that *S100A4* ^−/−^ might have advantages of eliminating the bacteria.

### Infection-induced colitis and colonic pathology are attenuated in S100A4-deficient mice

**Figure 3:**
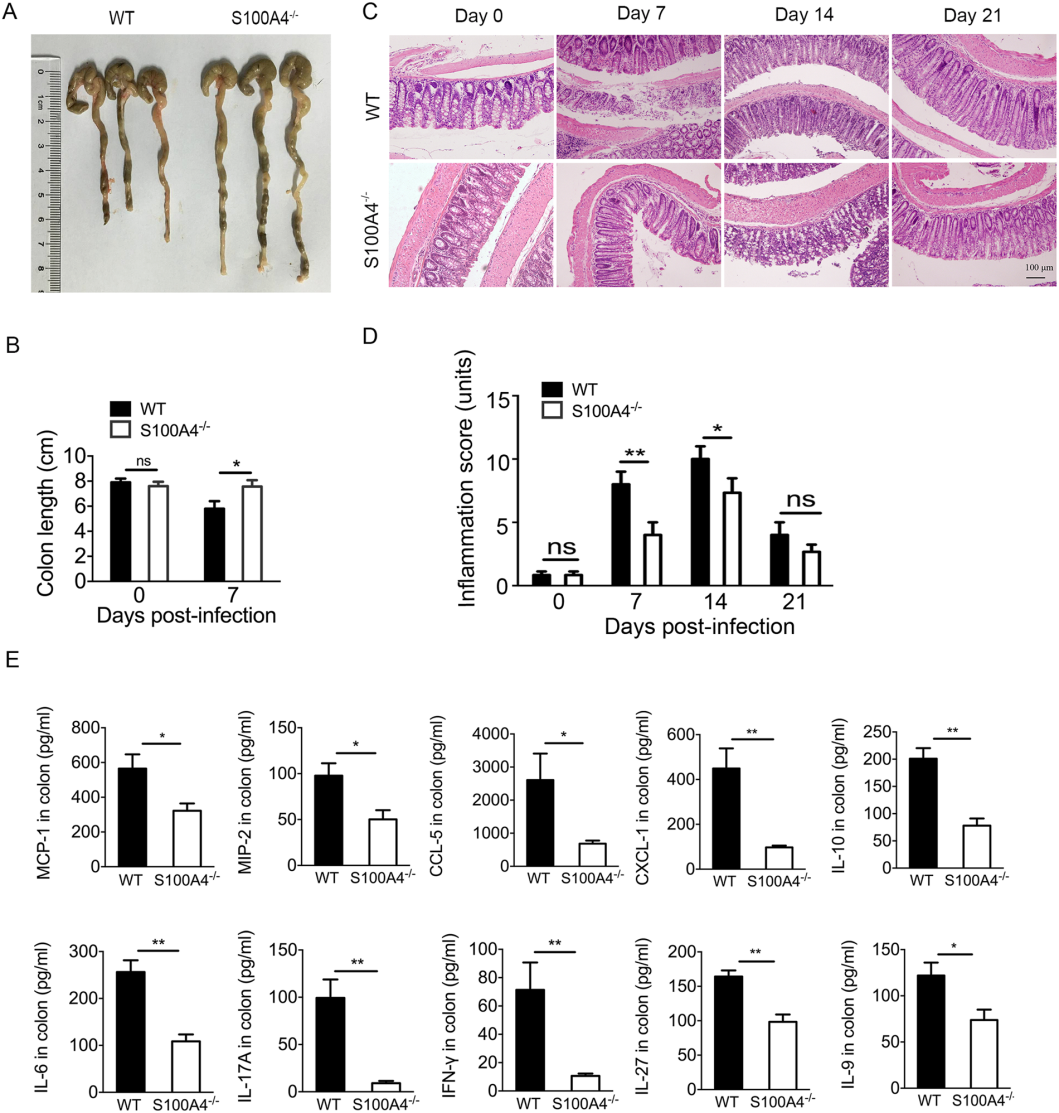
Infection-induced colitis and colonic pathology are attenuated in S100A4-deficient mice. Groups of WT and *S100A4* ^−/−^ mice were infected orally with *C. rodentium*. (**A**) Representative colon figures from WT and *S100A4* ^−/−^mice (*n* = 3) on day 7 after infection. (**B**) Colon length from WT and *S100A4* ^−/−^ mice (*n* = 3) on day 0 and day 7 after infection; *P < 0.05. (**C**) On day 0 (uninfected), day 7, 14, and 21 after infection, colons were sectioned and stained with H&E (*n* = 3). (**D**) Inflammation scores of WT mice and *S100A4* ^−/−^ mice colon on day 0, 7, 14 and 21 p.i. The combined score equals the sum of the separate scores, including Edema in the sub-mucosa, PMN infiltration, reduced number of goblet cells and ulcerate epithelial layer, (*n* = 3); *P < 0.05, **P < 0.01. (**E**) Protein levels of some chemokines and inflammatory cytokines in the colons of WT and *S100A4* ^−/−^ on day 7 p.i. with *C*. *rodentium* (*n* = 4), *P < 0.05, **P < 0.01.

A number of previous studies have characterized *C*. *rodentium* infection in mice as causing significant intestinal inflammation and severe colonic pathology^25^. To characterize the pathology associated with *C*. *rodentium* infection in *S100A4* ^−/−^ mice, colons were removed from WT and *S100A4* ^−/−^ mice and evaluated for both macroscopic and microscopic appearance. The colon lengths in *S100A4* ^−/−^ mice were longer than in the WT mice on day 7 p.i. (*P* < 0.05) (Fig. 3A,B). Inflammation and tissue damage were observed in both WT and *S100A4* ^−/−^ mice, whereas there was a greater degree of inflammation in WT mice compared to the *S100A4* ^−/−^ mice (Fig. 3C). The combined inflammation scores in *S100A4* ^−/−^ mice were significantly attenuated compared to WT mice on day 7 *(P* < 0.01) and day 14 p.i. (*P* < 0.05) and were not significantly different on day 21 (Fig. 3D) when the infection began to clear. The individual inflammation score showed in Supplementary Fig. 4A, contained sub-mucosal edema, polymorphonuclear granulocytes (PMN), epithelial damage and goblet cell depletion.

Chemokines and cytokines are important mediators of inflammation. We therefore examined the gene expression of a series of chemokines and pro-inflammatory cytokines in WT and *S100A4* ^−/−^ mice infected with *C*. *rodentium*. As shown in Fig. 3E, colonic levels of the monocyte/macrophage chemokine MCP-1, the neutrophil chemoattractant MIP-2, CCL-5, CXCL-1 and pro-inflammatory cytokines interleukin-6 (IL-6), IL-17A, interferon gamma (IFN-γ) and IL-27 were decreased significantly in *S100A4* ^−/−^ mice compared to WT mice after *C*. *rodentium* infection on day 7 (*P* < 0.05, *P* < 0.01). In addition, the levels of anti-inflammatory cytokines IL-9 and IL-10 in *S100A4* ^−/−^ mice were also decreased compared to WT mice after *C*. *rodentium* infection on day 7 (*P* < 0.05, *P* < 0.01) (Fig. 3E). The mRNA expression of some inflammatory cytokines was also detected on day 14 and day 21. We found that the expression of MCP-1, MIP-2, IL-6 and IFN-γ were still down-regulated in *S100A4* ^−/−^ mice colon on day 14 (*P* < 0.01) and there were no differences on day 21, except IFN-γ (*P* < 0.01) (Supplementary Fig. 4B,C). These results demonstrate that S100A4 deficiency attenuates the overall immune response, specially decreased the colonic inflammation.

### S100A4 deficiency decreases inflammatory cell recruitment in the colon during *C*. *rodentium* infection

**Figure 4:**
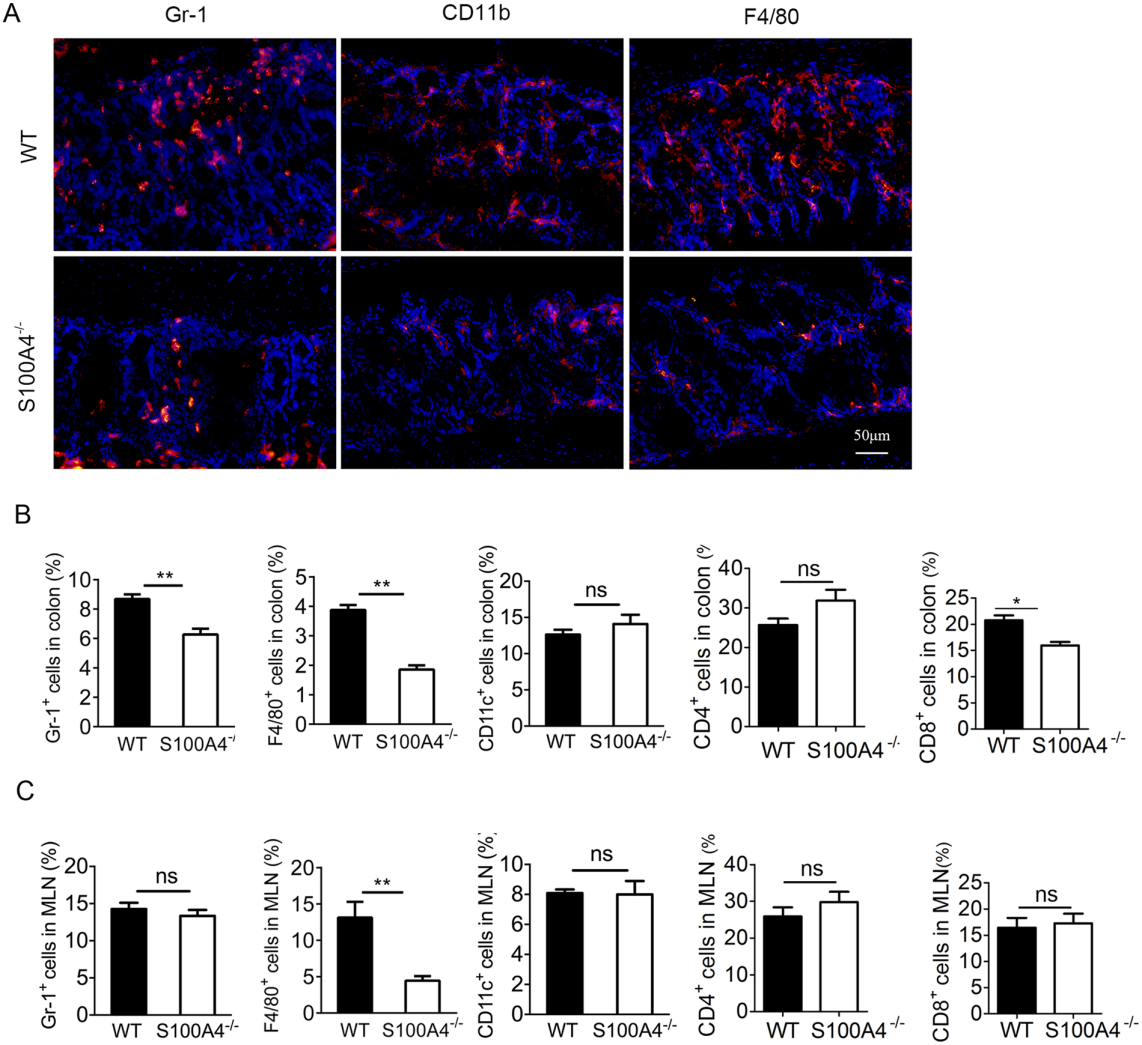
Early inflammatory cell recruitment during *C. rodentium* infection is S100A4 dependent. Groups of WT and *S100A4* ^−/−^ mice were infected orally with *C. rodentium*. (**A**) Colon sections stained with anti-Gr-1 (red), anti-CD11b (red) and anti-F4/80 (red) to detect neutrophil and macrophage infiltration on day 7 p.i. Representative images of the entire colon were shown (*n* = 3). The percentages of Gr-1^+^, F4/80^+^, CD11c^+^, CD4^+^, and CD8^+^, cells in colon (**B**) and MLN (**C**) from WT and *S100A4* ^−/−^ mice on day 7 p.i. (*n* = 3); **P < 0.01; ns, not significant.

MCP-1 and MIP-2 are important chemokines for the recruitment of macrophages and neutrophils^26^. We investigated whether the local inflammatory cell accumulation response to *C*. *rodentium* infection was influenced by S100A4 expression. By immunostaining, we found that a large number of macrophages and neutrophils accumulated in the infected colons using F4/80 and Gr-1 as the respective markers. Inflammatory cell infiltration was impaired in *S100A4* ^−/−^ mice compared to WT mice after *C*. *rodentium* infection (Fig. 4A). To further characterize the inflammatory cells’ response to *C*. *rodentium* infection, myeloid cells presented in the colonic lamina propria of WT and *S100A4* ^−/−^ mice were isolated and analyzed by flow cytometry. On day 7 after *C*. *rodentium* infection, cell percentages of all analyzed myeloid cell types (F4/80^+^, Gr-1^+^) in *S100A4* ^−/−^ mouse colons were significantly lower than those in WT colons (*P* < 0.01), except for that of CD11c^+^ cells (Fig. 4B). In addition, a dramatic decrease in percentage of F4/80^+^ macrophages was evident in the MLN of *S100A4* ^−/−^ mice (*P* < 0.01) (Fig. 4C). T cells presented in the colonic lamina propria and MLNs of WT and *S100A4* ^−/−^ mice were also isolated and analyzed by flow cytometry. On day 7 after *C*. *rodentium* infection, there were no significant differences of percentages of CD4^+^ T cells both in colons and MLNs between *S100A4* ^−/−^ and WT mice. In addition, the percentages of CD8^+^ cells in *S100A4* ^−/−^ mouse colons were significantly lower than those in WT colons (Fig. 4B). However, there was no significant difference in the MLN of *S100A4* ^−/−^ mice compared with WT mice (*P* < 0.01) (Fig. 4C). Collectively, our data suggest that S100A4 may play a role in colonic infections by promoting the inflammatory cell recruitment and inflammatory responses to pathogenic bacteria.

### S100A4 deficiency decreases cell proliferation in the colon during *C*. *rodentium* infection

**Figure 5:**
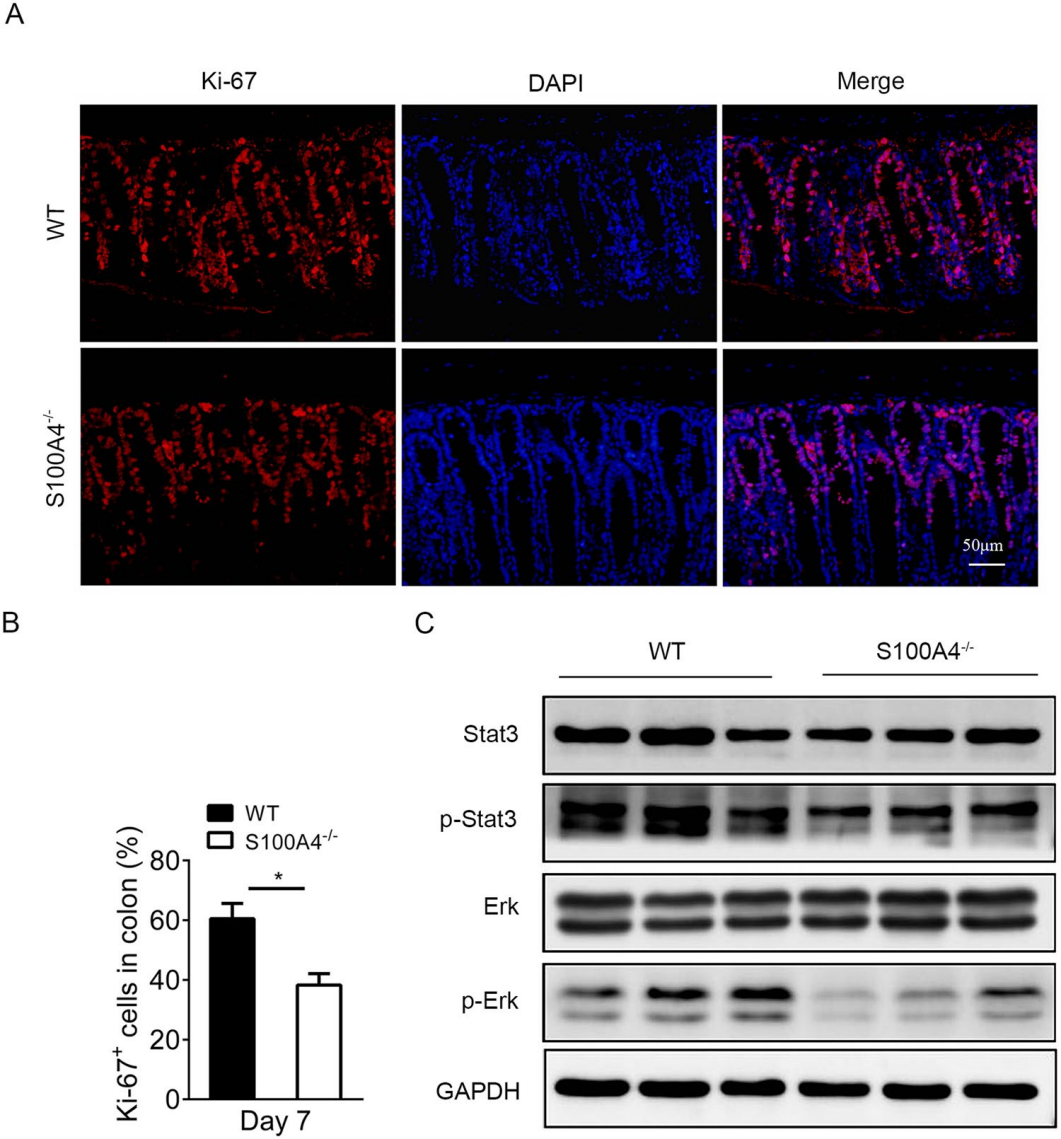
S100A4 deficiency decreases cell proliferation in the colon during *C. rodentium* infection. (**A**) On day 7 after infection, colons were sectioned and stained with anti-Ki-67 (red) and DAPI (blue). Representative images of the entire colon were shown (*n* = 3). (**B**) The percentages of Ki-67^+^ cells in colonic cells, (*n* = 3); *P < 0.05. The experiment was performed by an observer blinded to the experimental condition. (**C**) On day 7 after infection, protein levels of Stat3, Erk and phosphorylated Stat-3, Erk in colon tissues were detected by Western blot, (*n* = 3). GAPDH was used as the loading control. The full-length blots were presented in Supplementary Figure 2B.

As noted above, S100A4 deficiency resulted in decreased colonization and production of pro-inflammatory cytokines during *C*. *rodentium* infection. We examined the effect of *C*. *rodentium* infection on the expression of the proliferative marker Ki-67 in WT and *S100A4* ^−/−^ mice. As shown in Fig. 5A,B, there was a marked decrease in Ki-67- positive cells in the infected colon tissues of *S100A4* ^−/−^ mice compared with WT infected tissues on day 7 (*P* < 0.05). These results suggest that S100A4 promotes colonic cells proliferation during *C*. *rodentium* infection.

The expression of pro-inflammatory genes is modulated by signal transduction pathways such as NF-κB, Erk and Stat3. To determine whether these pathways and molecules were down-regulated in the absence of S100A4, we examined the activation of NF-κB, Stat3 and Erk signaling pathways by Western blot. We found that the p-Stat3, p-Erk levels in *C*. *rodentium* infected colon tissues in *S100A4* ^−/−^ mice were clearly down-regulated compared to those in WT mice (Fig. 5C, Supplementary Fig. 2B). The p-p65 level was also down-regulated in *S100A4* ^−/−^ mice (Supplementary Fig. 2A).

### S100A4 has no influence on peritoneal macrophages during infection with *C*. *rodentium ex vivo* and has no bactericidal ability

Previous studies have found that macrophages were intimately related with inflammation and the main effector cells playing an important role in bacterial infection^27^. Therefore, we determined the role of S100A4 during infection of primary macrophages. Peritoneal macrophages of WT and *S100A4* ^−/−^ mice were infected with *C*. *rodentium* and intracellular bacterial growth over time *ex vivo* was calculated. The bacterial counts at each time point were similar in WT and *S100A4* ^−/−^ mice (Supplementary Fig. 2A). We then tested the phagocytosis by peritoneal macrophages of WT and *S100A4* ^−/−^ mice *ex vivo*. The bacterial counts were detected after infection with *C*. *rodentium* at different time points. The lack of a difference in the results indicated that S100A4 does not affect the phagocytosis by macrophages *ex vivo* (Supplementary Fig. 2B).

Previous studies revealed that S100A4 has no direct effect on growth of *Staphylococcus aureus in vitro* ^28^. However, S100A15 (10 μg/ml) has antimicrobial activity against *E*. *coli* ^29^. To assess the bactericidal ability of S100A4, *C*. *rodentium* was incubated in Luria Broth at 37 °C with recombinant S100A4 (0–10 μg/ml). Viable counts (number of CFU) were calculated after 15 h and compared in S100A4-treated and untreated cultures. Bacterial counts in the S100A4-treated cultures were similar to those non-treated cultures at all concentration tested (Supplementary Fig. 2C), suggesting that S100A4 may have no bactericidal properties *in vitro*.

### S100A4 promotes adherence of *C*. *rodentium* to CT26 cells

**Figure 6:**
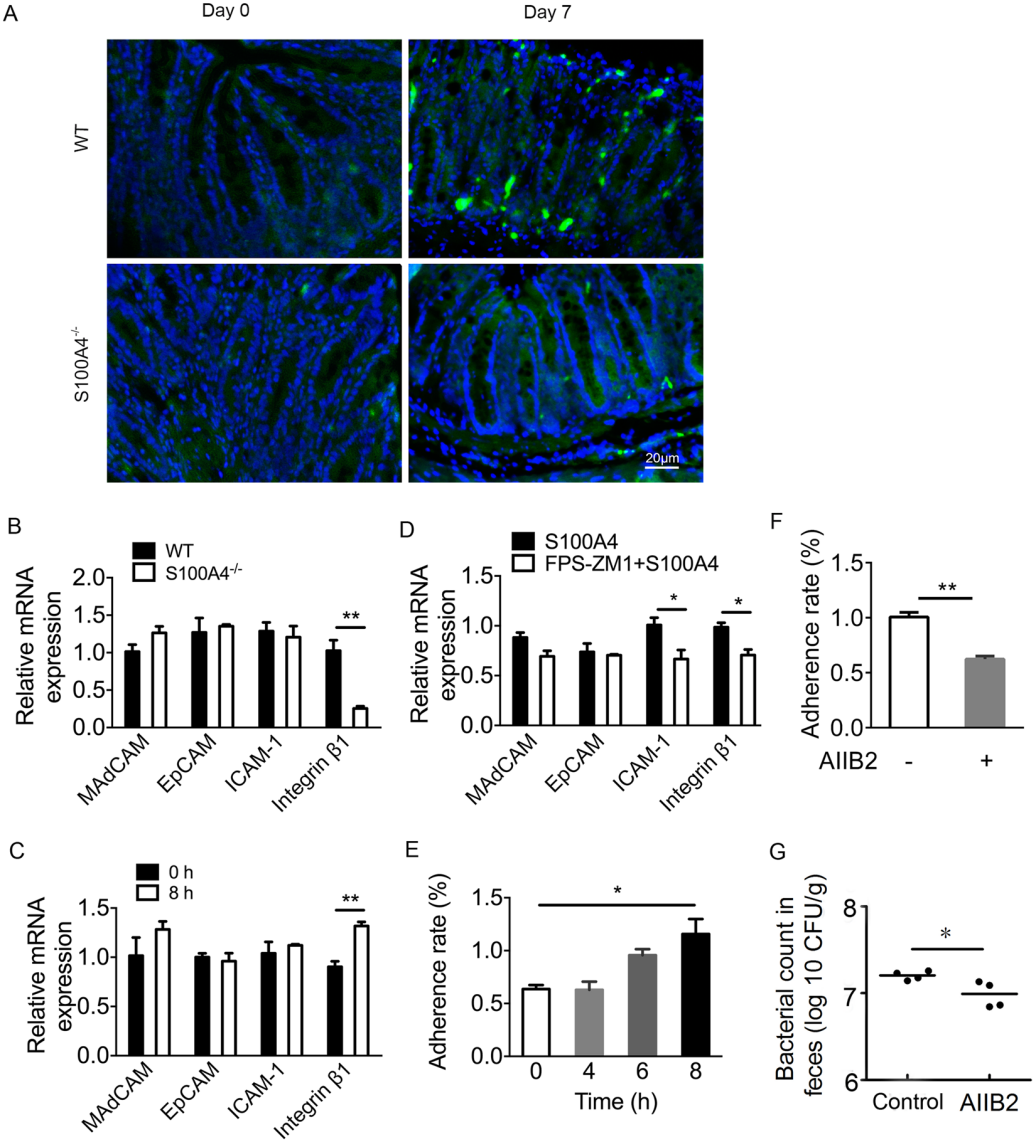
S100A4 increases *C. rodentium* adherence to CT26 cells. (**A**) FISH analysis of conventional WT and *S100A4* ^*−/−*^ mice colon on day 0 and day 7. Colon tissues were probed with a universal bacterial FISH probe (green) and counterstained with DAPI (blue), (scale bars, 20 μm). Representative images of the distal colon were shown (*n* = 3). (**B**) Real-time quantitative PCR analysis of the expression of mRNA encoding adhesion molecules in WT and *S100A4* ^*−/−*^ mice colons on day 7 p.i. GAPDH was used as the reference control, (*n* = 4); **P < 0.01. The mRNA level of WT mice is set as 1.00 to calibrate the relative level of *S100A4* ^−/−^ mice. (**C**) Real-time quantitative PCR analysis of the expression of mRNA encoding adhesion molecules in CT26 cells treated for 0 or 8 h with S100A4. GAPDH was used as the reference control, (*n* = 4); **P < 0.01. The mRNA level of 0 h is set as 1.00 to calibrate the relative level of 8 h. (**D**) Real-time quantitative PCR analysis of the expression of mRNA encoding adhesion molecules in CT26 cells treated for 0 or 8 hours with S100A4 after stimulating with FPS-ZM1 (10 μg/ml) for 1 h. GAPDH was used as the reference control, (*n* = 4); *P < 0.05. The mRNA level of cells that are not administrated with inhibitor FPS-ZM1 is set as 1.00 to calibrate the relative level of cells administrated with inhibitor FPS-ZM1. (**E**) The adherent ratios of *C. rodentium* to a CT26 cell monolayer were quantified by calculating CFU. CT26 cells were pretreated with S100A4 (1000 ng/ml) for 0–8 h before infection with *C. rodentium*, (*n* = 4); *P < 0.05. (**F**) The adherent ratio of *C. rodentium* to CT26 cells was quantified after stimulating with integrin blocker AIIB2 (2.5 μg/ml) for 1 h and S100A4 for 8 h, (*n* = 4); **P < 0.01. (**G**) Bacterial titers in fecal homogenates from AIIB2 treated WT mice and control WT mice on day 7 after *C*. *rodentium* infection (*n* = 5); *P < 0.05.

Adherence is important to bacterium for the colonization of intestinal epithelial cells. We characterized the localization of *C*. *rodentium* in colon tissue by fluorescence *in situ* hybridization (FISH) on day 0 and day 7 in WT and *S100A4* ^−/−^ mice after infection. The number of bacteria counts in lamina propria of *S100A4* ^−/−^ mice colon was decreased compared with that in WT mice colon on day 7 (Fig. 6A). In the process of adherence, whether some adhesion molecules were activated by S100A4 was unknown. Then, we used WT and *S100A4* ^−/−^ colons to detect the expression of genes encoding adhesion molecules, which are important for adhesion, such as integrin β-1, ICAM-1, EpCAM and MadCAM after infection with *C*. *rodentium*. We found that the expression of integrin β-1 in WT mice was significantly higher than *S100A4* ^−/−^ mice on day 7 (*P* < 0.01) (Fig. 6B). Because *C*. *rodentium* is a mouse pathogen, we choose mice colon adenocarcinoma cell line (CT26) to do researches *in vitro* ^30^. CT26 cells were treated with S100A4 for 8 h and the expression of genes encoding the same adhesion molecules were analyzed by real-time PCR. Indeed, the expression of integrin β−1 was significantly higher in the CT26 cells treated with S100A4 for 8 h (*P* < 0.01) (Fig. 6C). To further test whether this effect was S100A4 receptor RAGE dependent, we used N-Benzyl-4-chloro-N-cyclohexylbenzamide (FPS-ZM1), which can block RAGE, to treat CT26 cells for 1 h followed by stimulation with S100A4 for 8 h. The results showed that the expression of integrin β−1 was significantly decreased after treatment with the blocker (*P* < 0.05) (Fig. 6D). Furthermore, CT26 cells were treated with S100A4 for 0–8 h before infection with *C*. *rodentium* for 1 h. With the time extension, the adherence rate of *C*. *rodentium* to CT26 cells was increased (*P* < 0.05) (Fig. 6E), and the adherent ratio was remarkable higher at 8 h. To further demonstrate the role of integrin β−1 in the process of adherence, we used the blocker of integrin β−1, the AIIB2-anti β−1 integrin antibody, to suppress the function of integrin β−1. Indeed, the results showed that the adherent rate of *C*. *rodentium* to CT26 cells was significantly decreased after adding the integrin β−1 blocker (*P* < 0.01) (Fig. 6F). The effect of AIIB2 *in vivo* experiments has been detected. As shown in Fig. 6G, AIIB2 treated mice had significantly lower fecal counts of *C*. *rodentium* on day 7 p.i. (*P* < 0.05).The above results indicate that S100A4 can promote the adherence of *C*. *rodentium* to CT26 cells via regulating the expression of integrin β−1.

## Discussion

In this study, we demonstrated that S100A4 promotes colitis development via increasing the adherence of *C*. *rodentium* to intestinal epithelial cells. The expression of S100A4 was up-regulated in *C*. *rodentium*-infected mouse colons. *C*. *rodentium* infection induced weight loss, and its colonization was impaired in S100A4-deficient mice. Infection-induced colitis, colonic pathology and inflammatory cell recruitment were also attenuated in *S100A4* ^−/−−/−^ mice. Further studies revealed that S100A4 increased the adherence of *C*. *rodentium* to intestinal epithelial cells by promoting adhesion molecule integrin β-1 expression.

The infection of *C*. *rodentium* is used to model several important human intestinal diseases, such as Crohn’s disease and ulcerative colitis^31^. Mice infected with *C*. *rodentium* have become the recognized model for investigating the virulence mechanisms of A/E pathogens. During *C*. *rodentium* infection of the colonic epithelium, adherent bacteria translocate Tir (translocated intimin receptor) into the enterocyte by the type III secretion system (T3SS) and then integrate into the epithelium and form A/E lesions^32^. In the mucosal immune responses, *C*. *rodentium* can trigger inflammation due to the recognition of pathogen-associated molecular patterns (PAMPS), including lipopolysaccharide (LPS), and peptidoglycan. Subsequently, infection leads to the activation of the nuclear factor-κB (NF-κB) signaling pathway and the production of pro-inflammatory cytokines, such as IL-6 and tumor necrosis factor (TNF), using innate immune cells^33–35^. Previous studies have shown that S100A4 can regulate bacterial clearance and inflammatory responses^28^. Whether S100A4 influences *C*. *rodentium*-induced colitis was unknown. Here, in this study, we first demonstrated that S100A4 promotes bacteria-induced colitis, which elucidated a new mechanism of colitis pathology for *C*. *rodentium*.

S100A4 is involved in a variety of physiological functions, such as cell motility, adhesion, proliferation, invasion and metastasis^36^. Intracellularly, S100A4 binds to several targets regulating cytoskeletal dynamics and cell motility and proliferation. Moreover, S100A4 is secreted from both tumor and non-malignant cells and exerts extracellular effects regulating, in particular, angiogenesis, cell migration and cardiomyocyte differentiation^37^. In addition, S100A4 also has an important role in inflammation regulation. It stimulates cytokine production, particularly granulocyte colony-stimulating factor and eotaxin-2 from T lymphocytes^38^, and may thereby influence allergic inflammation as it is expressed in normal myeloid cells^39^. However, its role in host defense for bacterial infection has not been well established. It has been reported that certain members of the S100 family have bactericidal properties. The S100A8/S100A9 heterodimer inhibits microbial growth by depriving bacteria nutrients through metal chelation^23^. S100A7 can kill bacteria with a pH dependent target specificity^24^. However, S100A4 does not seem to play a protective role during infection. Bian *et al*. found that S100A4 contributed to bacterial accumulation at sites of infection and S100A4 deficiency is associated with efficient bacterial clearance during staphylococcal infection^28^. The impaired phagocytosis in *S100A4* ^−/−^ mice may reduce virulence by favoring bacterial killing. Our results demonstrated that the *C*. *rodentium* colonization of feces and tissues were attenuated in *S100A4* ^−/−^ mice. However, S100A4 was not bactericidal *in vitro* for *C*. *rodentium*. The mechanism of cleaning bacteria needs further research.

Monocytes and macrophages contribute to adaptive immune responses in the gut by producing cytokines that are essential for optimal Th1 polarization during *C*. *rodentium* infection^40^. Large numbers of macrophages were recruited to the infected intestine, and these inflammatory cells undoubtedly contribute to the tissue damage and disease pathology during infection. Prior to infection, we found only a small number of macrophages in the colon. We observed a rapid and dramatic recruitment of macrophages and neutrophils to the colons of WT mice on day 7 p.i. (Fig. 4A,B). While S100A4 deficient mice were infected similarly by large numbers of *C*. *rodentium*, we found few signs of macrophage or neutrophil recruitment in these mice, demonstrating a clearance that was S100A4 dependent for this early inflammatory response. In addition, conventional dendritic cells (cDCs) are essential to initiating adaptive T cell immunity in the gut after infection with *C*. *rodentium* ^41^. After infection, mice lacking GM-CSF had significantly fewer mucosal cDC, greater bacterial burden, increased mucosal inflammation, systemic spread of infection and delayed pathogen clearance. Furthermore, deletion of cDCs in CD11c DTR mice, led to weight loss and a fatal infection, demonstrating the essential role of cDCs to the immune response against *C*. *rodentium* and for controlling bacterial dissemination^40^. However, we did not found significantly decrease of cDC infiltration in *S100A4* ^−/−^ mice, suggesting that S100A4 maybe not obviously affect the recruitment of cDC after infection, and whether the function of cDC could be affected by S100A4 still need further study.

Levels of some chemokines and cytokines, such as MIP-2, MCP-1, IL-6, IL-17A, and IFN-γin colons were significantly attenuated in *S100A4* ^−/−^ mice after *C*. *rodentium* infection (Fig. 3E), which suggested reduced inflammation during early infection. In addition, the levels of IL-9 and IL-10 were also decreased in *S100A4* ^−/−^ mice. It has been reported that IL-9 functions in anti-inflammatory immune responses related to parasitic infection and allergic reactions^42^. However, recently, IL-9 was found to have a critical role in the pathogenesis of IBD. There was an association of IL-9 expression and IL-9^+^ T cells with the severity of pathology of ulcerative colitis^43^. Furthermore, in an animal model of IBD, IL-9-deficient T cells and IL-9 neutralization attenuated colitis^44^. The role of IL-9 in CR induced inflammatory regulation still need further study. IL-10 is a potent anti-inflammatory cytokine essential for protecting the host against excessive inflammatory and immune responses^45^. It was reported that a subset of macrophages in colon that produces IL-10 plays a critical role in preventing excessive inflammation following acute CR infection by limiting innate immunity^46^. However, other study reported that IL-10 is not required for limiting inflammation in response to *C*. *rodentium* ^47^. IL-10-deficient mice had less acute infection-associated colitis and cleared infection faster than wild-type controls, which is consistent with our results. Therefore, its immune functions may be context dependent. The colonization of *C*. *rodentium* in epithelial cells was significantly decreased in *S100A4* ^−/−^ mice. All of the pathological features that we assessed were attenuated in *S100A4* ^−/−^ mice, suggesting that S100A4-mediated inflammatory changes may facilitate *C*. *rodentium* colonization in the colon. Understanding how A/E bacterial pathogens colonize their hosts is of great importance in combating these infections. More studies are needed to establish how inflammation may facilitate *C*. *rodentium* colonization of the host gut.

Adherence is an essential step in bacterial pathogenesis and infection and is required for colonization in a new host. Attachment of bacteria to cell surfaces protects them from elimination by host bactericidal proteins and serves as an important virulence mechanism^48^. There are six families of eukaryotic cell adhesion molecules (CAMs), including the immunoglobulin-like superfamily, the cadherins, the integrins, the receptor protein tyrosine phosphatases, the selectins and the hyaluronate receptors^49^. CAMs are used by various microorganisms, including EPEC. Adherent bacteria translocate Tir into the enterocyte by T3SS. In addition, β−1 integrin may act as an alternative receptor^50–52^. Some studies have demonstrated that EPEC and EHEC reinforces adherence to the host cell by interacting with integrin linked kinase (ILK). The interaction between ILK and bacterial effectors increased cell surface levels of β−1 integrin^53^. In our study, we found that S100A4 could up-regulate the mRNA expression of integrin β−1 in CT26 epithelial cells. As a result, *C*. *rodentium* increased adherence to CT26 cells. However, *C*. *rodentium* decreased adherence to CT26 cells when added the integrin β−1 blocker. How S100A4 interacts with integrin β−1 still needs further study.

In conclusion, our results demonstrate that *C*. *rodentium* has profited from S100A4, to enhance host adhesion and colonization through the S100A4-mediated host inflammatory responses. A lack of S100A4 results in impaired inflammation and *C. rodentium* colonization and increased protection against *C*. *rodentium* induced colitis. This finding provides direct evidence and a novel explanation for how S100A4 influences bacteria infectious colitis. S100A4 may provide a promising strategy for treatment of colitis.

## Materials and Methods

### Mice

*S100A4* ^−/−^ mice on a C57BL/6 background and S100A4^+/+^.GFP transgenic mice were purchased from the Jackson Laboratory (Bar Harbor, ME). All of the mice were maintained under specific pathogen free conditions in the animal facilities at the Institute of Biophysics, Chinese Academy of Sciences. *S100A4* ^−/−^ mice and littermate controls used for experiments were 6- to 8-weeks-old. This study was carried out in strict accordance with the recommendations in the Guide for the Care and Use of Laboratory Animals of the Chinese Academy of Sciences. The experiments described were approved by the Institutional Animal Care and Use Committee of Institute of Biophysics, Chinese Academy of Sciences.

### Bacterial strains and infection of mice

The *C*. *rodentium* strain DBS100 (ATCC 51459; American Type Culture Collection) was grown by shaking overnight in Luria-Bertani broth at 37 °C. *S100A4* ^−/−^ mice and WT mice were orally inoculated with 2 × 10^9^ CFU *C*. *rodentium* in a total volume of 200 μl per mouse. Body weights were assessed at the beginning of infection and every 2 days after infection.

For integrin β−1 function-blocking experiment, integrin β−1 blocking AIIB2 antibody (2 mg/kg) or nonspecific rat IgG was injected into the i.p. cavity of WT mice biweekly on day 1 and day 4 after *C*. *rodentium* infection. Bacterial titers in fecal homogenates from AIIB2 treated WT mice and control WT mice on day 7 after *C*. *rodentium* infection were detected.

### Reagents and antibodies

Recombinant S100A4 protein (10185-H01H) was obtained from Sino Biological Inc (Beijing, China). Antibodies against p65 (4764 S), phosphorylated p65 (3036 S), Erk (4695 S), phosphorylated Erk (4370 S), Stat3 (12640 S) and phosphorylated Stat3 (9145 S) were obtained from Cell Signaling Technology (Beverly, MA). Peroxidase-conjugated goat anti-mouse or goat anti-rabbit secondary antibodies were purchased from Huaxingbio Biotechnology Co. (Beijing, China). Immunohistochemical antibodies were obtained from BD Pharmingen (Franklin Lakes, New Jersey). The S100A4 (GR208945-1) antibody was obtained from Abcam (Cambridge, MA), and the F4/80 (122601) antibody was obtained from Biolegend (San Diego, CA). Antibodies for flow cytometry were all obtained from Biolegend (San Diego, CA). Integrin β−1 function-blocking antibody AIIB2 was obtained from Millipore (Temecula, CA).

### Tissue collection, bacterial counts and histopathological analysis

Colons were obtained from *S100A4* ^−/−^ mice and WT mice. Some colons were fixed with 4% paraformaldehyde while others were fixed with optimum cutting temperature compound (O.C.T.). Paraffin-embedded tissues were stained with hematoxylin and eosin (H&E) and S100A4 and those embedded in O.C.T. were stained for inflammatory cells. Mesenteric lymph nodes, livers, spleens, colons and feces were removed aseptically then weighed and homogenized in PBS. Homogenates were diluted and plated on MacConkey agar plates. *C*. *rodentium* colonies were pink with white rings. The inflammation score was calculated using a previously described scoring system^54^. The inflammation combined score from adding all the measures listed in the Barthel article, contained submucosal edema, neutrophil infiltration, epithelial damage and goblet cell depletion. Pathological evaluation was performed by an observer blinded to the experimental condition.

### Isolation of Lamina Propria Cells

Colons were dissected, rinsed with ice-cold PBS supplemented with antibiotics (penicillin plus streptomycin), and cut into small pieces. Colons pieces were then incubated with RPMI medium supplemented with 3% FBS, 0.5 mM DTT, 5 mM EDTA, and antibiotics at 37 °C for 30 min with gentle shaking. After removing the epithelial layer, the remaining colon segments were incubated at 37 °C with RPMI medium containing 0.5% Collagenase D (Roche) and 0.05% DNase (Roche) for 30 min with gentle shaking. Then, the supernatant was passed through a 70 μm cell strainer and the lamina propria cells were isolated on a 40/80 Percoll gradient.

### Real-time quantitative PCR

Total RNA extracted from mouse tissues and CT26 cells with Trizol reagent (CWBIO biotech Co., Beijing) were reverse transcribed with a PrimeScript RT Reagent kit (TaKaRa, Dalian, China). Quantitative real-time PCR (qPCR) was performed using a SYBR Premix ExTaq II (TaKaRa) and the ABI-Prism 7500 Sequence Detection System (Applied Biosystems). The expression of target genes was normalized to expression of housekeeping gene GAPDH. The 2^−ΔΔCT^ method was used to determine the fold changes in mRNA levels of each sample, as compared with the reference sample.

### Cytokine analysis

To detect multiple cytokines in the colons tissues were homogenized in ice-cold TE buffer. Homogenates were centrifuged at 12,000 × g for 15 minutes. The supernatant was collected, and the ProcartaPlex^TM^ multiplex immunoassay (Luminex) (eBioscience) was used on a Bioplex-200 system with the Bioplex Manger 5.0 software. The cytokines were analyzed according to the manufacturer’s protocol.

### Flow cytometry analysis

Single-cell suspensions prepared directly from colon mesenteric lymph nodes and spleens were stained with the following directly labeled mouse-specific mAbs: PE labeled anti-Ly6C (clone HK1.4), APC labeled anti-F4/80 (clone BM8), and PE labeled anti-CD11c (clone N418). Cells were collected on a FACSCalibur (BD Biosciences, San Diego, CA) and analyzed by FlowJo software (TreeStar, Ashland, OR).

### Western blot analysis

Colon extracts were analyzed by Western blotting as described^55^. The primary antibodies used were anti-stat3, anti-p-stat3, anti-p65, anti-p-p65, anti-Erk, anti-p-Erk, and anti-GAPDH. Horseradish peroxidase (HRP)-conjugated goat anti-mouse or goat anti-rabbit were used as the secondary antibodies. The chemiluminescent signal was detected by using Super Enhanced Chemiluminescence Kit (Huaxingbio, Beijing).

### Bactericidal properties of S100A4 *in vitro*

*C*. *rodentium* was incubated in Luria Broth at 37 °C with recombinant S100A4 (0–10 μg/ml). Diluted *C*. *rodentium* were plated on the MacConkey agar. Viable counts (number of CFU) were calculated after 15 h.

### The survival of *C*. *rodentium* and the phagocytosis by macrophages *ex vivo*

Peritoneal macrophages of WT and *S100A4* ^−/−^ mice were collected by peritoneal lavage. Cells were pooled together from two mice. For *C*. *rodentium* growth curves, cells were plated in 24-well plates at a density of 8 × 10^5^ cells/ml and grown until confluent. Peritoneal macrophages were infected with *C*. *rodentium* at a multiplicity of infection (MOI) of 10 for 0.5 h. Fresh medium was added to the cells with 50 μg/ml gentamicin after washing 3 times with PBS. At each time point, the cell monolayers were rinsed 3 times and then lysed with 0.1% saponin on ice for 20 min. The number of bacteria was calculated by counting the colonies on MacConkey agar plates. In the phagocytosis assay, the density of peritoneal macrophages and *C*. *rodentium* was the same as the survival assay. At each time point, the cell monolayers were washed 3 times with PBS and then lysed with 0.1% saponin on ice for 20 min. The number of phagocytic bacteria was counted similar to *C*. *rodentium* as described previously.

### Adherence assay

CT26 cells were cultured in 24-well plates at a density of 4 × 10^5^ cells/ml and grown until confluent. The cell monolayers were stimulated with S100A4 for 0–8 h. Then, log-phase *C*. *rodentium* was resuspended in RPIM-1640 medium without antibiotics and diluted to 2 × 10^6^ CFU/ml, and then added to CT26 cells at an MOI of 5 for 1 h at 37 °C in 5% CO_2_. The cells were lysed with 0.1% saponin on ice for 20 min after washing 3 times with PBS. The number of adherent bacteria was counted by plating on MacConkey agar plates.

### FISH Analysis

Colons were prepared by fixation in Carnoy’s fixative (Ricca Chemical) and embedded in paraffin. Tissues were sectioned at 5-μm thicknesses and hybridized to a bacterial 16 S rRNA gene probe. Hybridizations were performed as described^56^ and visualized under a fluorescence microscope (DP71, OLYMPUS).

### Statistical analysis

All of the data were expressed as the mean ± SEM and analyzed using GraphPad Prism software. Differences between two groups were compared using a two-tailed unpaired Student’s t-test for parametric data analysis. Non-parametric data were analyzed using One-way ANOVA followed by Tukey’s test. For all tests, a P values < 0.05 was considered statistically significant.

## Electronic supplementary material

supplementary information
