# Supplementary material for: S100A4 contributes to colitis development by increasing the adherence of Citrobacter rodentium in intestinal epithelial cells
Source: Sci Rep. 2017 Sep 21;7:12099. doi: 10.1038/s41598-017-12256-z (PMC5608709; doi:10.1038/s41598-017-12256-z)
Supplement: Supplementary file 1 — supplementary information [file 41598_2017_12256_MOESM1_ESM.doc]

S100A4 contributes to colitis development by increasing the adherence of *Citrobacter rodentium* in intestinal epithelial cells

Jinhua Zhang^1, †^, *, Ying Jiao^2, †^, Shasha Hou^1^, Tian Tian^1^, Qi Yuan^1^, Huaijie Hao^3^, Zhenlong Wu^4^, Xuexiang Bao^2^*

**Supplementary figure 1**

**S100A4 is expressed in different types of cells in colon after *C. rodentium* infection.**


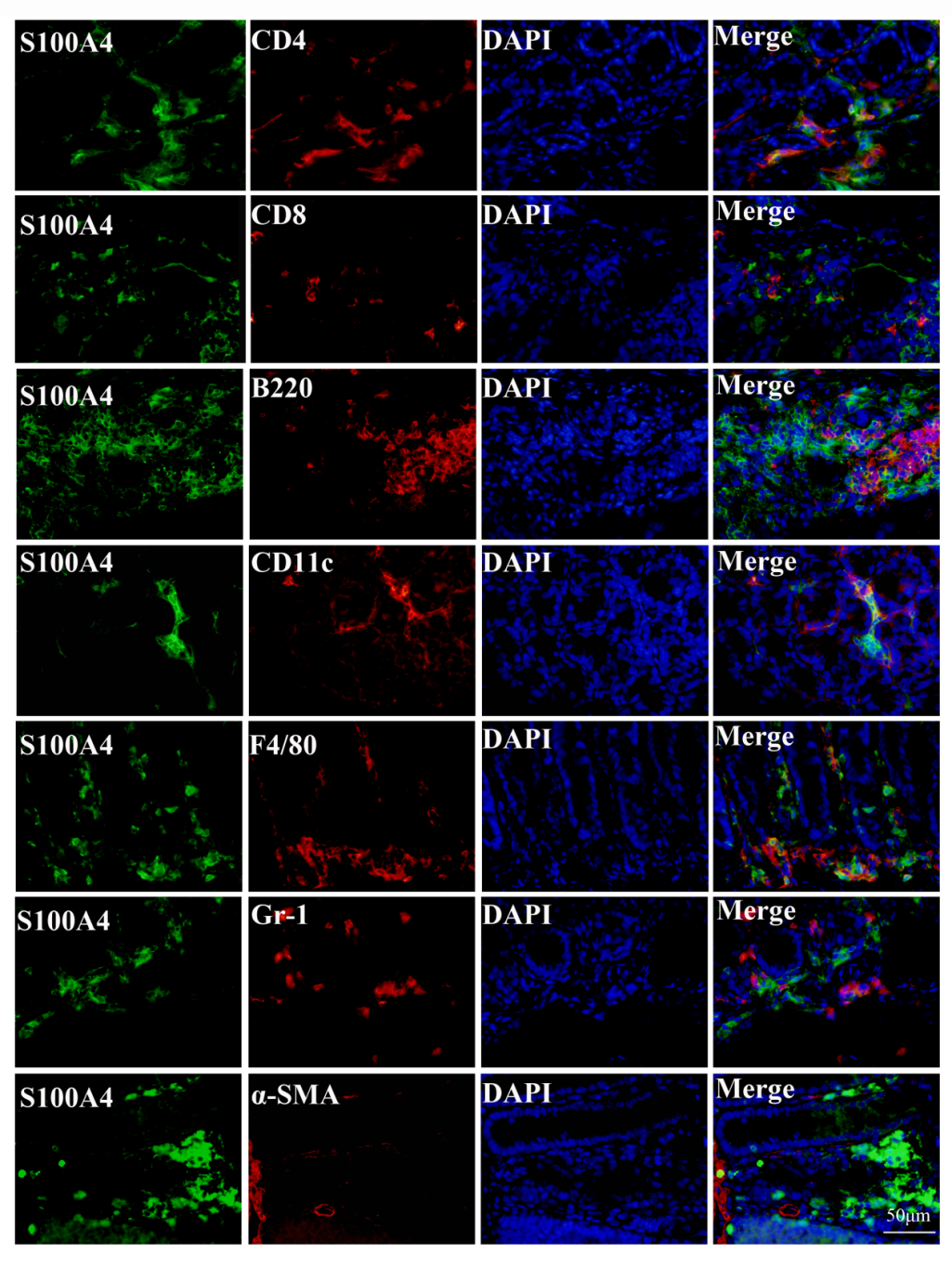


Double immunofluorescence staining for S100A4 (Green) and different cell markers, CD4, CD8, B220, CD11c, Gr-1 and α-SMA (Red) in colon sections from C57BL/6 mice on day 7 after *C. rodentium* infection (*n* = 4).

**Supplementary figure 2**

**S100A4 does not affect the growth of *C. rodentium* in peritoneal macrophages and the phagocytosis by macrophages *ex vivo*.**


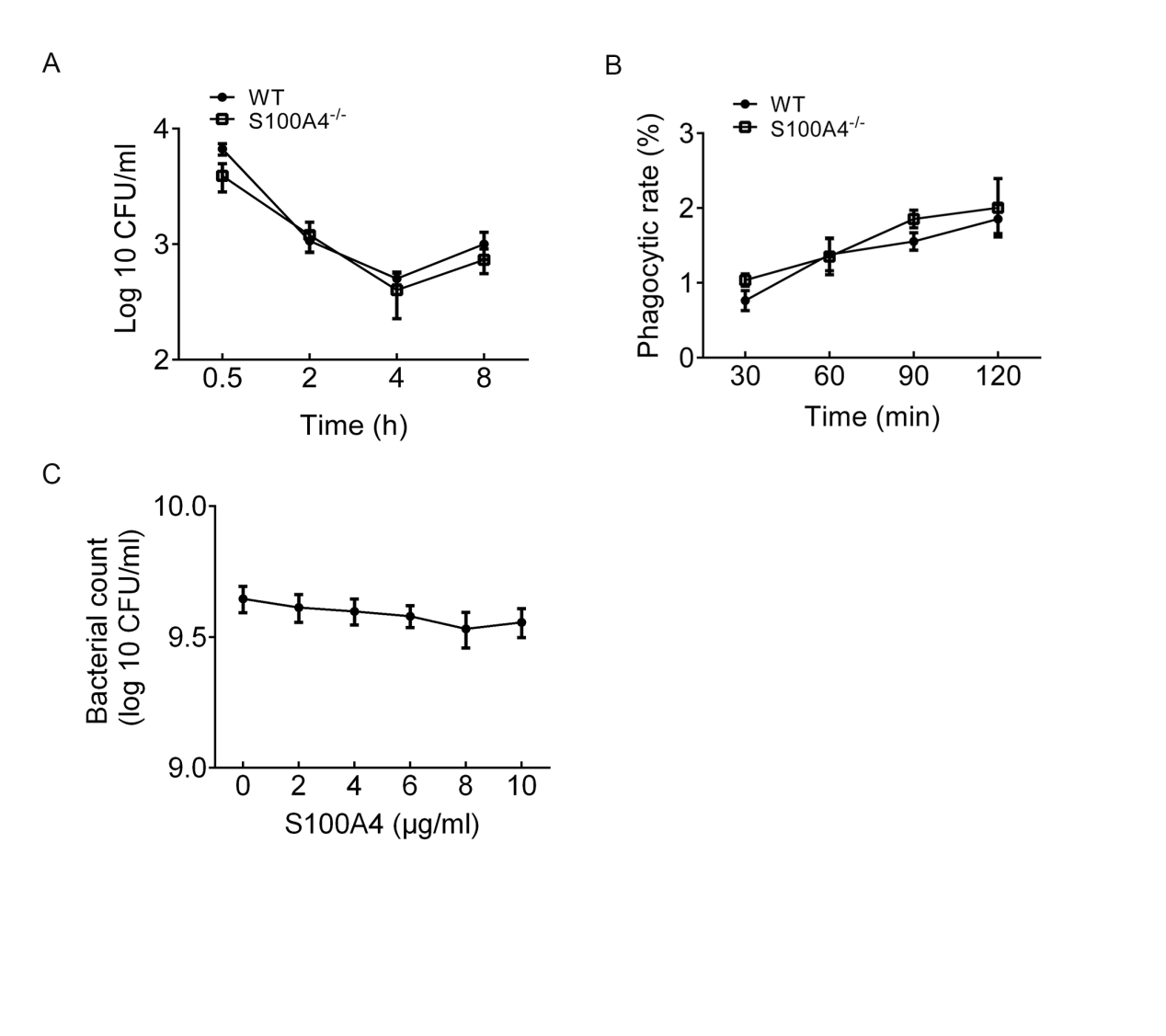


(A) Intracellular growth of *C. rodentium* in peritoneal macrophages of WT and *S100A4^-/-^* mice. Peritoneal macrophages were infected with an MOI of 10 (*n*=4), *P* > 0.05. (B) The phagocytic rate of peritoneal macrophage to *C. rodentium* was detected at the different time points (*n*=4), *P* > 0.05. (C) The bactericidal assay of S100A4 (0-10 μg/ml) to *C. rodentium in vitro* (*n*=3), *P* > 0.05*.*

**Supplementary figure 3**


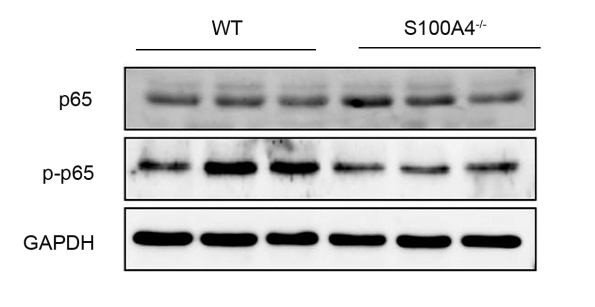
A


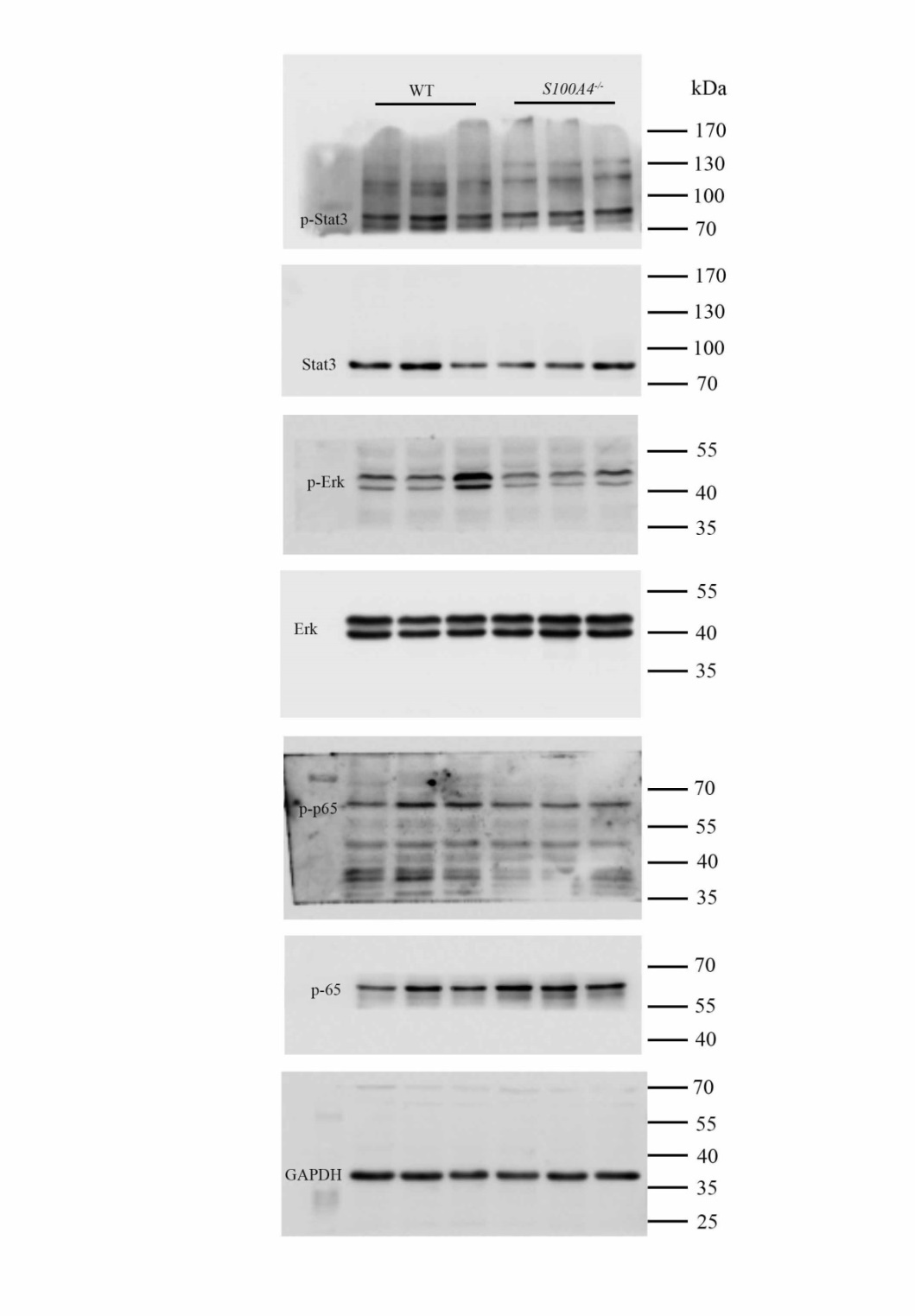
B

(A) On day 7 after infection, protein levels of p-65 and phosphorylated p-65 in colon tissues were detected by Western blot, (*n* = 3). GAPDH was used as the loading control. (B) The origin data of these panels is identical with figure 5 showing Western blot with stat3, p-stat3, p65, p-p65, Erk, p-Erk and GAPDH antibodies.

**Supplementary figure 4**


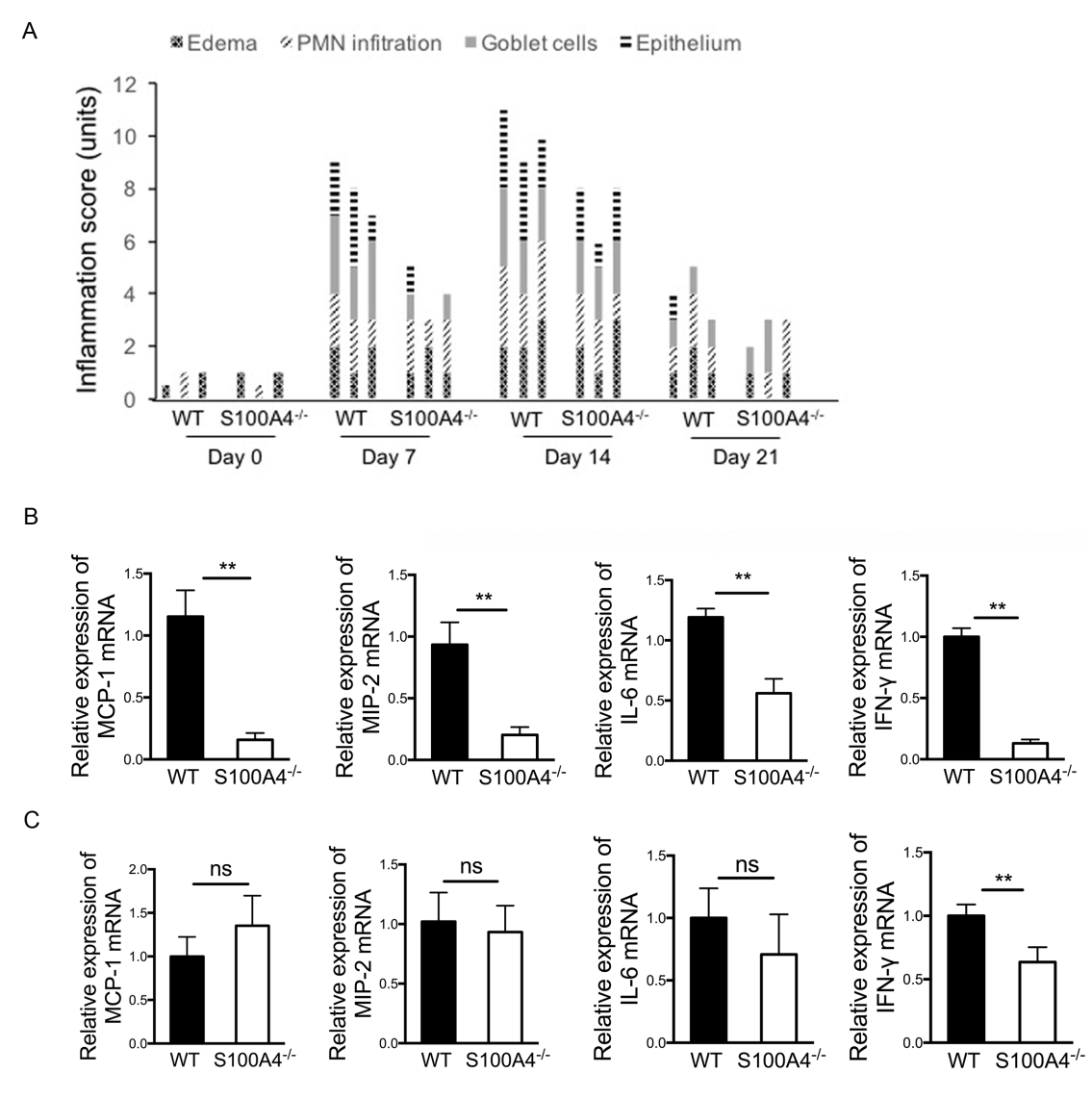


(A) Scoring of inflammatory changes on day 0, day 7, day 14, day 21 p.i. of WT and *S100A4^-/-^* mice. We scored H&E-stained sections of the colons from three mice from each group. Edema in the sub-mucosa, PMN infiltration, reduced number of goblet cells and ulcerate epithelial layer were scored individually. Real-time quantitative PCR analysis of some chemokines and inflammatory cytokines in colons from infected WT mice and *S100A4^-/-^* mice on day 14 (B) and day 21 (C). GAPDH was used as the reference control, (*n* = 4); ***P* < 0.01. The mRNA level of WT mice is set as 1.00 to calibrate the relative levels in *S100A4^-/-^* mice.
